# Free sugar intake from snacks and beverages in Canadian preschool- and toddler-aged children: a cross-sectional study

**DOI:** 10.1186/s40795-023-00702-3

**Published:** 2023-03-08

**Authors:** Jessica Yu, Anisha Mahajan, Gerarda Darlington, Andrea C. Buchholz, Alison M. Duncan, Jess Haines, David W. L. Ma, Guelph Family Health Study

**Affiliations:** 1 Department of Human Health and Nutritional Sciences, University of Guelph, 50 Stone Road East, Guelph, ON N1G 2W1 Canada; 2 Department of Mathematics and Statistics, University of Guelph, 50 Stone Road East, Guelph, ON N1G 2W1 Canada; 3 Department of Family Relations and Applied Nutrition, University of Guelph, 50 Stone Road East, Guelph, ON N1G 2W1 Canada

**Keywords:** Preschoolers, Toddlers, Free sugar, Snacks, Beverages, Recommendations, Dietary patterns, ASA24-Canada

## Abstract

**Background:**

Excess consumption of free sugar (FS) increases the risk of dental caries and unhealthy weight gain. However, the contribution of snacks and beverages to young children’s FS intake is not well understood. The purpose of this study was to determine FS intake from snacks and beverages among preschool-aged Canadian children.

**Methods:**

This cross-sectional study examined baseline data from 267 children 1.5 to 5 y enrolled in the Guelph Family Health Study. Dietary assessment was completed over a 24-h period using ASA24-Canada-2016 to, 1) estimate the proportion of children whose FS intake from snacks and beverages consumed exceeded 5% total energy intake (TE) and 10% TE, and 2) identify the top snack and beverage sources of FS.

**Results:**

FS contributed 10.6 ± 6.9% TE (mean ± SD). 30 and 8% of children consumed ≥ 5% TE and ≥ 10% TE from snack FS, respectively. Furthermore, 17 and 7% of children consumed ≥ 5% TE and ≥ 10% TE from beverages FS, respectively. Snacks and beverages accounted for 49 ± 30.9% of FS energy. Top snack sources of FS (% children, children’s %TE from FS) were bakery products (55%, 2.4%), candy and sweet condiments (21%, 3.0%), and sugar-containing beverages (20%, 4.1%). Top sugar-containing beverage sources of FS (48%, 5.3%) were 100% fruit juice (22%, 4.6%) and flavored milk (11%, 3.1%).

**Conclusions:**

Snacks and beverages contributed nearly half of FS intake among a sample of young children in Canada. Thus, long-term monitoring of snacking behavior and consumption of FS is warranted. These findings may help inform nutritional strategies and public policies to improve diet quality and FS intake in preschool-aged children.

**Trial registration:**

The Clinical Trial Registry number is NCT02939261 from clinicaltrials.gov. Date of Registration: October 20, 2016.

**Supplementary Information:**

The online version contains supplementary material available at 10.1186/s40795-023-00702-3.

## Background

The World Health Organization (WHO) recommends that free sugar (FS) intake be limited to < 10% of total energy (TE) to reduce the risk of unhealthy weight gain and < 5% of TE to reduce the risk of dental caries [1]. According to the WHO, FS is defined as monosaccharides and disaccharides added to foods and beverages by the manufacturer, cook or consumer, and sugars naturally present in honey, syrups, fruit juices and fruit juice concentrates [1]. Studies investigating preschool- and toddler-aged children have found that 70–96% of children exceed the WHO free sugar intake recommendation of < 5% TE [2–6], which is of concern because of short term consequences such as dental caries and since dietary habits formed in young children have been found to track into adulthood [1, 7].

Canada’s new dietary guidelines [8] have adopted the WHO recommendation that FS intake be limited to < 10% TE. Overall reduction of FS through several strategies including the preparation of nutritious snacks with little to no added sugars and the selection of water as the beverage of choice is encouraged [8]. However, the contribution of snacks and beverages to FS intake remains poorly understood, which is concerning because snacking is becoming more prevalent and contributes > 25% TE in children living in Canada and the United States (US) [9–13]. Studies in these countries reveal promising data that sweet beverage consumption is decreasing in children; however, intake still remains high [14, 15]. Continuous monitoring is necessary given evidence that FS intakes from beverages have particularly adverse impacts on cardiovascular disease risk [1, 16].

While it is known that many young children exceed recommendations for FS, few studies have explored specific food or beverage intake and sources of FS [2, 4, 5]. Understanding the top sources of FS can help inform approaches to reduce sugar intake among young children. This study investigated the contribution of snacks, beverages, and their categories (e.g., bakery products, frozen desserts, candy and sweet condiments, flavored milk, 100% fruit juice) to FS intake among preschool-aged Canadian children. The objectives of the present study were to investigate the proportion of children whose FS intakes exceed < 5% TE and < 10%TE through snacks and beverages; and to identify leading snack and beverage sources of FS.

## Methods

### Ethics approval and consent to participate

The present study adhered to the guidelines of the Declaration of Helsinki and was approved by the University of Guelph Research Ethics Board (REB#17-07-003). Parents provided written informed consent.

### Study design

This is a cross-sectional secondary analysis of baseline data obtained from children participating in the Guelph Family Health Study-Full Study cohort. Details describing the study design and recruitment have been published elsewhere [17]. The aim of this study was to investigate FS intake from snack and beverage categories among preschool-aged children.

### Setting and participants

The Guelph Family Health Study is an ongoing family-based behavior change intervention study (NCT02939261). Between 2017–2020, families with at least one child between the ages of 1.5 and 5 y and who were not planning to move in the next year were recruited from Guelph-Wellington areas via the Guelph Family Health Team, Guelph Community Health Centre, community events, and social media platforms.

### Dietary assessment

A single 24-h dietary assessment was completed at baseline for each child by one parent using the online Automated Self-Administered 24-h (ASA24) Dietary Assessment Tool 2016-Canadian version (National Cancer Institute, Bethesda, MD). The Canadian version of ASA24 calculates the nutrient profile of reported dietary intake using the Food Patterns Equivalents Database (FPED), Food and Nutrients Database for Dietary Studies (FNDDS), and Canadian Nutrient File (CNF). Energy and nutrient intakes were screened for outliers using the adjusted box plot method [18] and implausible dietary intakes.

Total sugar and added sugar intakes were calculated by ASA24. FS intake was defined as added sugar plus sugar from 100% fruit juice (includes fruit juice concentrate diluted to single strength and fruit juice that is not from concentrate) [1, 19]; and determined using a standardized and semi-automated stepwise approach as described in Additional File 1.

### Snacks, beverages, and their categories

Snacks were defined as foods (i.e., food snacks) and beverages excluding water (i.e., beverage snacks) consumed between parent-identified meals as per ASA24 meal occasion coding. This is consistent with previous research investigating snacking in preschool-aged children using 24-h recall [13, 20, 21]. Beverages were defined as all beverages (including water) consumed at any meal or snack during the 24-h recall. Items were classified into snack and beverage categories by two data analysts to ensure quality of data entry. Snack and beverage items were classified into 15 categories and several subcategories using a classification system adapted from Bernstein et al. [22] (See Additional file 2). These categories included sugar-focused major food groups created based on the Canadian Food and Drug Regulation’s Schedule M food categories [22]. FS intakes were determined from these sources.

### Statistical analyses

Statistical analyses were completed using SAS® University Edition version 9.4 (SAS Institute, Inc., Cary, NC). The percent contributions (mean ± SD) of snacks, beverages, and their FS to TE and total FS energy were estimated. These data were used to determine the proportion of children whose FS intakes exceeded < 5% TE and < 10% TE from snacks or beverage alone.

To identify snack and beverage sources of FS, the proportion of children consuming each snack and beverage category (See Additional file 2) and each category’s FS contribution to TE were determined. The frequency and percent of children consuming each snack and beverage category (regardless of serving size) over 24-h were reported. For each participant, the energy consumed from FS per snack and beverage category relative to TE (%TE from FS) was determined by summing FS energy for the snack or beverage category, dividing by the participant’s TE and then multiplying by 100. Next, participants’ %TE from FS (mean, 95% CI) was calculated for snack and beverage categories consumed by at least 30 participants (to ensure adequate data for a meaningful summary). Generalized estimating equations were used to account for any dependence between sibling participants [23].

## Results

### Sample characteristics

A total of 322 children were enrolled in the Guelph Family Health Study full study cohort. Data were excluded from ASA24 records that were incomplete (*n* = 28), included breastmilk (*n* = 15) or had sugar intakes that were considered both an outlier (less than the 25^th^ percentile minus 1.5 times the IQR or greater than the 75^th^ percentile plus 1.5 times the IQR) and implausible (*n* = 12). Thus, the final cross-sectional analysis included baseline data from 267 (*n* = 129 boys, *n* = 138 girls) children from 210 families. Additional file 3 presents a participant flow chart. In the final analytical sample, over half (52%) of families had a household annual income of ≥ $100,000 Canadian and the majority (79%) had at least one parent with a university degree or higher (Table 1). Most families (73%) had one child included in the analysis. The majority of children (77%) were White with an average age of 3.6 ± 1.2 y (mean ± SD). TE in the sample of children was summarized as 1411 ± 385 kcal/d (mean ± SD).

**Table 1:** Demographic characteristics of families and participants

**Family Characteristics (*n* = 210)**	**n (%)**
**Household Income (Canadian Dollars)**	
< $60,000	33 (16)
$60,000 – $99,999	56 (27)
≥ $100,000	110 (52)
Did not answer	11 (5)
**Highest level of education obtained by at least one parent**	
Some university, some college or technical school, high school graduate	13 (6)
College graduate	31 (15)
University graduate	63 (30)
Postgraduate training or degree	103 (49)
**Child(ren) included in this analysis**	
1 child	154 (73)
2 or 3 children	56 (27)
**Participant Characteristics (*n* = 267)**	**n (%)**
**Child Ethnicity**	
White	205 (77)
Other	55 (21)
Did not answer	7 (3)
**Child Age in years,** Mean ± SD	3.6 ± 1.2
**Child Sex**	**N (%), Mean age (years) ± SD**
Male	129 (48), 3.6 ± 1.3
Female	138 (52), 3.5 ± 1.2
**Child Total Energy Intake in kcal,** Mean ± SD	1411 ± 385

### Snacks and beverages: free sugar energy

Among all children, FS contributed an average of 10.6% of TE (Table 2). Furthermore, FS from snacks and beverages contributed an average of 5.7% of TE, which is half of all energy contributed by different sources of FS. Individually, snacks contributed approximately one-third and beverages amounted to one-fifth of FS energy.

**Table 2:** Percent of children’s total energy contributed by free sugar, snacks, and beverages^a^

	% TE (mean ± SD)	% total FS energy (mean ± SD)
**Total FS**	10.6 ± 6.9	-
**FS from snacks**	3.9 ± 4.0	36.5 ± 28.1
FS from food snacks	3.1 ± 3.4	29.9 ± 26.7
FS from beverage snacks	0.8 ± 2.2	6.6 ± 16.4
**FS from beverages**	2.6 ± 4.2	19.3 ± 26.0

*FS* Free sugar, *TE* total energy

^a^Sample included (*n* = 267) non-breastfed Canadian children 1.5–5 years from the Guelph Family Healthy Study. Total FS is of all meals, snacks, and beverages consumed over 24-h. Beverage snacks are beverages (excluding water) consumed during snacking occasions (i.e., between meals), whereas beverage items, including water and FS from beverage snacks, were consumed any time within the 24-h recall

### Food snacks, beverage snacks, and free sugar energy

**Fig. 1:**
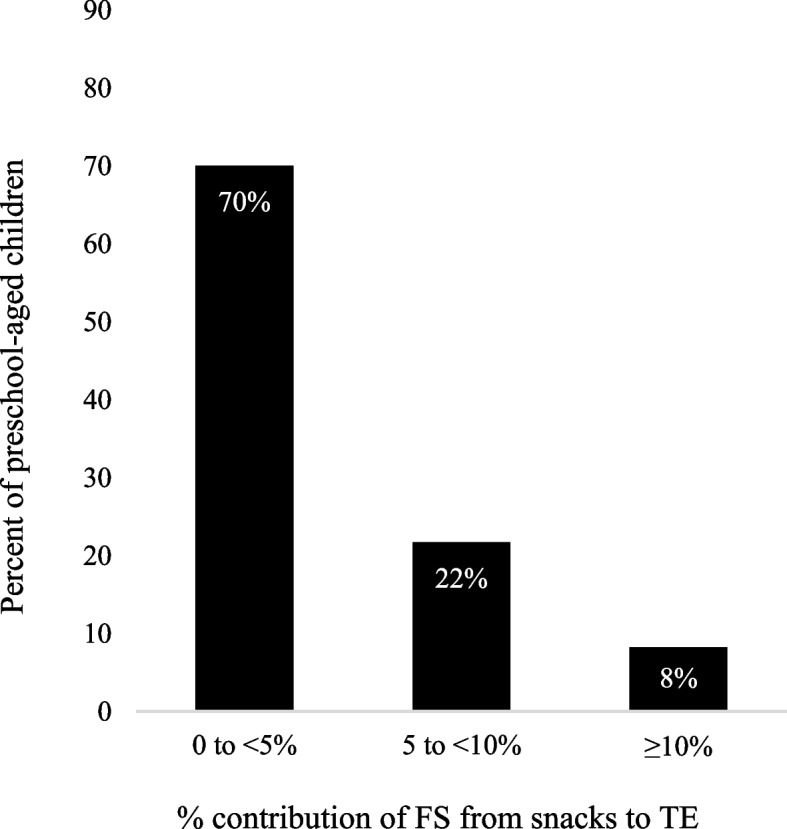
Percent of children consuming 0 to < 5, 5 to < 10 or ≥ 10% of TE from snack free sugar. Sample included (*n* = 267) Canadian children 1.5-5y. The ≥ 10% group ranges from 10 to 23% of TE. FS, Free Sugar; TE, Total Energy

Almost all (97%) children consumed snacks and most (91%) consumed ≥ 2 snack items (Table 3). An additional table shows all snack categories assessed (See Additional File 4). Thirty percent and 8% of children consumed ≥ 5% TE and ≥ 10% TE from snack FS, respectively (Fig. 1).

**Table 3:** Snack intake by snack categories and their contributions to free sugar energy intake among children^a^

Snack Category	Number of Children (Percent)			Children’s %TE from FS Mean (95% CI)
	≥ 1	1	≥ 2	
**ALL SNACKS**	260 (97)	17 (6)	243 (91)	4.0 (3.5 – 4.5)
**BEVERAGE SNACKS**	121 (45)	81 (30)	40 (15)	1.8 (1.3 – 2.3)
Sugar-Containing Beverages	53 (20)	41 (15)	12 (4)	4.1 (3.2 – 4.9)
Plain Milk	73 (27)	49 (18)	24 (9)	0
**FOOD SNACKS**	257 (96)	24 (9)	233 (87)	3.2 (2.8 – 3.6)
Candy and Sweet Condiments	56 (21)	50 (19)	6 (2)	3.0 (2.3 – 3.8)
Bakery Products	148 (55)	108 (40)	40 (15)	2.4 (2.1 – 2.7)
Dairy Products and Alternates	94 (35)	69 (26)	25 (9)	1.1 (0.7 – 1.6)
Savory Snacks	111 (42)	93 (35)	18 (7)	0.3 (0.2 – 0.5)
Fruits	191 (72)	86 (32)	105 (39)	0.5 (0.3 – 0.8)
Vegetables and Legumes (except fried potatoes)	44 (16)	30 (11)	14 (5)	0.01 (0.0 – 0.03)
Cereals and Grain Products	29 (11)	24 (9)	5 (2)	-
Nuts and Seeds	42 (16)	35 (13)	7 (3)	0.1 (0.1 – 0.2)
Mixed Dishes, Sides and Entrees	16 (6)	~	~	-
Frozen Desserts	12 (4)	~	~	-
Meats, Eggs and Substitutes	11 (4)	~	~	-

*FS* Free Sugar, *TE* Total Energy

^a^This table summarizes the number and percent of children 1.5–5 years who consumed different snack categories, relative to the total number of children (*n* = 267). Percent was calculated to identify the proportion of children who consumed snack categories at least once (≥ 1) over 24-h, then calculated for the more discrete categories of one time (1) or two or more times (≥ 2). Not all children were reported to consume a snack (*n* = 7). Mean children’s %TE from FS was calculated for categories that were consumed by ≥ 30 children. For these calculations, only data from children who consumed each respective category were used (i.e., data from children who did not consume the snack category over 24-h were not included in the mean). Generalized estimating equations were used to account for any dependence between sibling participants [23]. For categories consumed by < 30 children, mean %TE from FS was not calculated and was instead denoted with a dash. Categories consumed and/or breakdowns were not reported and were denoted as ~ whenever < 5 children were identified. Sugar-containing beverages included beverages with sugars added during processing + 100% fruit juice. An expanded version of this table including food and beverage items included in each snack category can be found in Additional File 4

Among children who consumed snacks, snack FS contributed 4.0% TE. Although food snacks (96%) were consumed by more children than beverage snacks (45%) and food snacks contributed more FS than beverage snacks, the single snack category that contributed the most %TE from FS was sugar-containing beverages (SCBs) followed by candy and sweet condiments, and bakery products. These categories were also consumed by large proportions of children: 20% for SCBs, 21% for candy and sweet condiments, and 55% for bakery products. Health Canada recommends that most sugars come from fruit, vegetables, and unsweetened dairy products [8, 24]. The proportions of children consuming these snacks were 72% for fruits, 27% for plain milk, 17% for cheese, 16% for vegetables and legumes, and 4% for plain yogurt.

### Beverages and free sugar energy

**Fig. 2:**
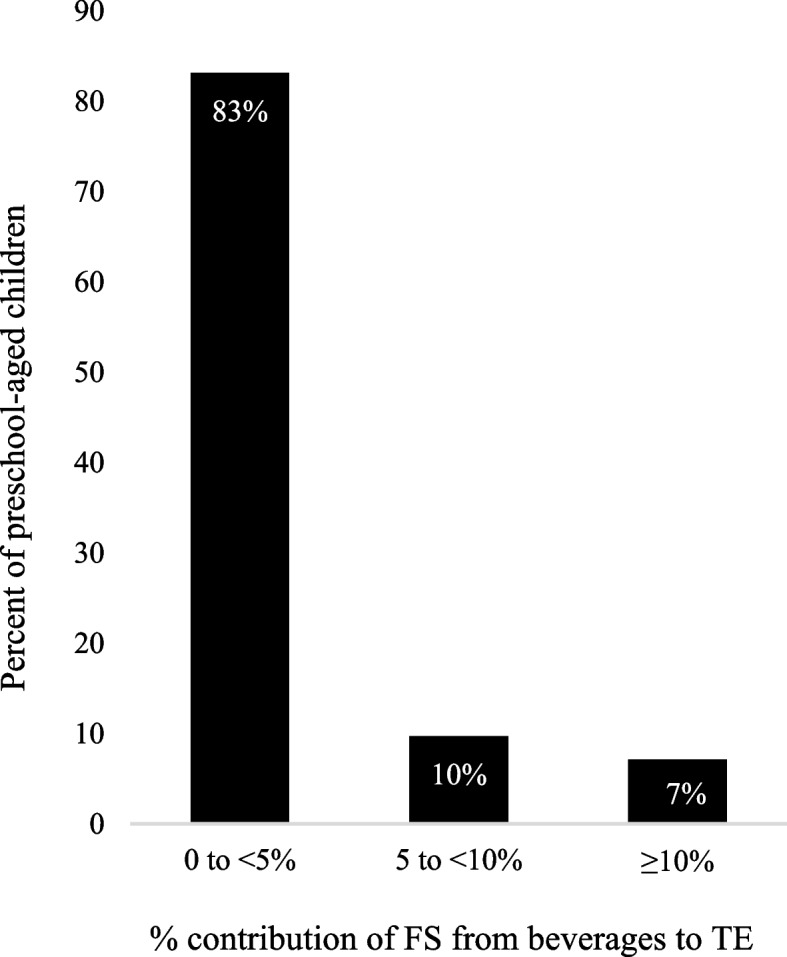
Percent of children consuming 0 to < 5, 5 to < 10 or ≥ 10% of TE from beverage free sugar. Sample included (*n* = 267) Canadian children 1.5-5y. The ≥ 10% group ranges from 10 to 26% of TE. FS, Free Sugar; TE, Total Energy

In this sample, 17 and 7% of children consumed ≥ 5% TE and ≥ 10% TE from beverage FS, respectively (Fig. 2). Almost all (99%) children consumed beverages ≥ 2 times over 24-h (Table 4).

**Table 4:** Beverage intake by beverage categories and their contribution to free sugar energy intake among children^a^

Beverage Category	Number of Children (Percent)			Children’s %TE from FS Mean (95% CI)
	≥ 1	1	≥ 2	
All Beverages	265 (99)	2 (0.7)	263 (98.5)	2.6 (2.1 – 3.1)
Sugar-Containing Beverages	129 (48)	79 (30)	50 (19)	5.3 (4.5 – 6.1)
100% Fruit Juice	58 (22)	48 (18)	10 (4)	4.6 (3.7 – 5.6)
Flavored Milk	29 (11)	~	~	3.1 (2.3 – 4.0)
Water	245 (92)	31 (12)	214 (80)	0
Plain Milk	180 (67)	66 (25)	114 (43)	0
Smoothies	23 (9)	~	~	-
Plant-Based Beverages	21 (8)	15 (6)	6 (2)	-
Fruit Drinks	16 (6)	~	~	-
Yogurt Beverages	15 (6)	~	~	-
Hot Beverages	7 (3)	~	~	-
Regular Soft Drink	~	~	~	-
Sports Drinks	~	~	~	-
Diet Soft Drink	0	0	0	-
Energy Drinks	0	0	0	-
Vegetable Drinks	0	0	0	-

^a^This table summarizes the number and percent of children 1.5–5 years who consumed different beverage categories, relative to the total number of children (*n* = 267). Percent was calculated to identify the proportion of children who consumed beverage categories at least once (≥ 1) over 24-h, then calculated for the more discrete categories of one time (1) or two or more times (≥ 2). Not all children were reported to consume a snack (*n* = 7). Not all children were reported to consume a beverage (*n* = 2). Mean children’s %TE from FS was calculated for categories that were consumed by ≥ 30 children. For these calculations, only data from children who consumed each respective category were used (i.e., data from children who did not consume the beverage category over 24-h were not included in the mean). Generalized estimating equations were used to account for any dependence between sibling participants [23]. For categories consumed by < 30 children, mean %TE from FS was not calculated and was instead denoted with a dash. Categories consumed and/or breakdowns were not reported and were denoted as ~ whenever < 5 children were identified. Sugar-containing beverages include beverages with sugars added during processing + 100% fruit juice. In this sample, this includes flavored milk, smoothies, sweetened plant-based beverages, fruit drinks, yogurt beverages, sweetened hot beverages, and 100% fruit juice

*FS* Free Sugar, *TE* Total Energy

SCBs, consumed by nearly half (49%) of children, included 100% fruit juice, flavored milk, smoothies, sweetened plant-based beverages, fruit drinks, flavored yogurt beverages, sweetened hot beverages, regular soft drinks, and sports drinks. More children consumed SCBs once (30%) compared to multiple times (19%) over 24-h. FS from SCBs contributed a mean of 5.3% TE. The SCB that was consumed by the highest proportion of children and contributed the greatest %TE from FS among children was 100% fruit juice followed by flavored milk. Smoothies, plant-based beverages, fruit drinks, yogurt beverages, and hot beverages were each consumed by fewer than 8% of children. Regular soft drinks and sports drinks were rarely consumed. Diet soft drinks, energy drinks, and vegetable drinks were not reported in any ASA24-records. Water and plain milk were the beverage categories consumed by the largest proportions of children (92 and 68%, respectively) and were more commonly consumed multiple times than once over 24-h.

## Discussion

In this study, we determined the proportion of children whose FS intake from snacks and beverages exceeds 5% TE and 10% TE, respectively; and identified leading snack and beverage sources of FS. Overall, we found that FS contributed 10.6% TE and that snacks and beverages combined contributed nearly half of all FS energy. To understand the breakdown of FS sources, examination revealed that 30 and 8% of children exceeded 5% TE and 10% TE through snack FS, respectively. We acknowledge that beverages are not only consumed during snacking occasions, but also throughout the day. Therefore, we completed further examination of FS from beverages, which showed that 17 and 6% of children exceeded 5% TE and 10% TE through beverages, respectively. Both snack and beverage analyses reveal excess contribution to FS intake. Finally, SCBs, bakery products, and candy and sweet condiments were the top snack and beverage sources of FS energy.

The WHO and Health Canada recognize snacks and beverages as potential areas to reduce FS intake and improve diet quality [1, 8]. Snacking is increasing in young children and the present study found that snacks contributed over one-quarter (26.6%) of TE, similar to preschoolers from Canadian (27%) and US (28%) national datasets [9, 12, 13]. FS energy as a proportion of TE was lower in our study (10.6%) than a recent analysis of children 1–8 y from Canadian Community Health Survey (CCHS)-2015 (13.8%) [4] but still exceeded 10% TE [1]. The findings that SCBs, bakery products, and candy and sweet condiments were the top snack sources of FS are consistent with previous studies [4, 5] and provide new information that these sources are commonly consumed during snacking occasions. SCBs are of particular concern due to their association with cardiometabolic risk [1, 16]. Despite reductions in SCB intake among young Canadian children [15], our findings that SCBs (particularly 100% fruit juice and flavored milk) were consumed by nearly half of the children and contributed FS greater than 5% TE reinforces that SCBs are still a concern among preschoolers and toddlers.

Possible strategies to reduce FS from snacks and beverages include replacing with healthy alternatives, reducing frequency of consumption, and reducing portion size. Replacing top snack and beverage sources of FS with fruits and vegetables during snacking occasions can limit FS intake and improve diet quality in young children, as demonstrated by Reale et al. [25]. Health Canada recommends a healthy eating pattern where most sugars come from fruit, vegetables, and unsweetened dairy products, items, which contain no FS [8, 24]. In our study, fruits and plain milk are snack and beverage categories that were consumed by a large proportion of children. Substituting sweetened items such as flavored yogurt with plain yogurt plus unsweetened fruits, and flavored milk with plain milk, can reduce FS intake while retaining the nutrients these items offer. Reducing portion size and frequency of consumption of sweetened snacks and beverages may also reduce FS intake. A previous study of a sample of 26 US preschoolers found that providing children with a larger size of beverage as a snack increased beverage and/or food intake and serving 100% fruit juice led to greater overall snack energy intake [26]. Given that SCBs were mostly consumed once a day among children in our study, portion size of beverages may be a larger contributor of excess FS intake than frequency of consumption. Furthermore, our data suggest that children who consume SCBs during snack time, consume more FS from that snack category than from any other snack category (Table 3).

Some limitations should be considered when interpreting our study results. Most participants came from middle-income households, had educated parents, and were White. Given that household income, parental education and ethnicity have been associated with diet quality and sweet snack and beverage intake, our findings may not be generalizable to children of diverse backgrounds [9, 27, 28]. To reduce participant burden, FS was calculated from a single day of dietary recall, which may vary from usual intake, and thus cannot be directly compared to WHO FS recommendations. Finally, using ASA24-Canada-2016 to calculate FS resulted in the use of data from multiple databases (i.e., FPED, CNF-2015, FNDDS) with sometimes inconsistent added sugar and total sugar values. In particular, “muffins with fruits/and or nuts” and “lemonade” are items where FS sometimes exceeded total sugar, suggesting an overestimation of FS in these items although, of note, these items were consumed by 11 and 5 children, respectively. Despite these potential overestimations, the contribution of FS to TE was lower in our study compared to children from the CCHS-2015 (10.6% TE in 1.5 – 5 y versus 13.8% TE in 1–8 y children), which analyzed FS using a different methodology [4].

Strengths of this study include the focus on how snack and beverage categories contribute to FS intake among young Canadian children. Furthermore, this study assessed FS as a percentage of TE, a measure used by various health agencies [1, 8].

## Conclusions

In this sample of young Canadian preschool-aged children, nearly half of FS energy intake came from snacks and beverages. Our study provides evidence that even at a young age, children consume snacks and beverages containing FS throughout the day at levels higher than currently recommended. We also identified the major sources of FS including bakery products, candy and sweet condiments, 100% fruit juice, and flavored milk. These findings help to inform nutrition recommendations and potential policy to address the contribution of sugar in the diet quality of preschool-aged children.

## Supplementary Information

**Additional file 1.** Stepwise determination of free sugars from ASA24-Canada-2016 data.

**Additional file 2.** Categories and subcategories of snacks and beverages (Adapted from Bernstein et al., 2016).

**Additional file 3.** Participant flow chart.

**Additional file 4.** Snack intake by major and minor snack categories and their contribution to free sugar energy intake among children.

**Additional file 5.** Guelph Family Health Study consortium members.

## Data Availability

The GFHS welcomes external collaborators. The datasets generated and/or analysed during the current study are not publicly available due to Research Ethics Board Restrictions but are available from the corresponding author, David W. L. Ma at davidma@uoguelph.ca on reasonable request.

## Abbreviations

ASA24: Automated Self-Administered 24-h
CCHS: Canadian Community Health Survey
CNF: Canadian Nutrient File
FPED: Food Patterns Equivalents Database
FNDDS: Food and Nutrient Database for Dietary Studies
FS: Free sugar
WHO: World Health Organization
TE: Total energy
SCB: Sugar-containing beverage
