# Supplementary material for: Free sugar intake from snacks and beverages in Canadian preschool- and toddler-aged children: a cross-sectional study
Source: BMC Nutr. 2023 Mar 8;9:44. doi: 10.1186/s40795-023-00702-3 (PMC9996946; doi:10.1186/s40795-023-00702-3)
Supplement: Supplementary file 1 — Additional file 1. Stepwise determination of free sugars from ASA24-Canada-2016 data. [file 40795_2023_702_MOESM1_ESM.pdf]

Additional File 1: Stepwise determination of free sugars from ASA24-Canada-2016 data (.pdf)

1	**Added Sugar (tsp. eq.) was converted to kcal**
	a. Added sugar (tsp. eq.) from foods and beverages was determined by ASA24.<br>b. Added sugar (tsp. eq.) was converted to kcal using the following equation: [1]Added Sugar (kcal) = Added Sugar (tsp. eq.) x (4.2 g/1 tsp. eq). x (4 kcal/1 g).
2	**'Sugar from Fruit Juice' was calculated for foods and beverages**
	a. Food and beverage items containing 100% fruit juice were identified using the ASA-24 F_JUICE variable. Items were categorized as 100% fruit juice or Mixed Items [2].<br>b. For 100% fruit juice (with no added sugar), free sugar is equal to total sugar and so the ASA24 total sugar data were used.<br>c. For Mixed Items, the type of 100% fruit juice in the food or beverage item was identified. If it could not be reasonably identified, apple juice was used since it is the most common fruit juice.<br>a. Sugar from Fruit Juice was calculated for each food and beverage item using the following equation: Sugar from Fruit Juice = Fruit juice (cups.eq.) x Grams Sugar Per Cup Fruit Juice[3].<br>b. For each food and beverage item, sugar from fruit juice was converted from grams to kcal using the following equation: Sugar from fruit juice (kcal) = Sugar from fruit juice (g) x 4 kcal / 1g
3	**Free Sugar (kcal) was calculated**
	a. Free sugar was calculated for each food and beverage item by summing 'Added Sugar' (Step 1) and 'Sugar from Fruit Juice' (Step 2).

[1]ASA24 calculates added sugar using the USDA's Food Patterns Equivalents Database (FPED) definition of added sugars. This includes all sugars added during processing, honey, syrups, and sugars from undiluted fruit juice concentrates (Bowman et al., 2018). The World Health Organization defines free sugar as monosaccharides and disaccharides added to foods and beverages by the manufacturer, cook or consumer, and sugars naturally present in honey, syrups, fruit juices and fruit juice concentrates (WHO, 2015). ASA24 added sugars were calculated based on the FPED definition of added sugars where one teaspoon equivalent of added sugars was reported to be equivalent to 4.2 grams.

[2] ASA24 items (Food_Description) from the full study baseline children cohort that contain fruit juice were categorized into 1) 100% Fruit Juice or 2) Mixed Items. 1) 100% Fruit Juice includes 100% fruit juice & Fruit juice concentrate diluted to single strength with no added sugar. Items in this category included "Fruit juice blend, 100% juice, with added Vitamin C", "Orange juice, chilled, includes from concentrate", "Pomegranate juice, ready-to-drink", "Apple juice, canned or bottled, added vitamin C", "Fruit juice, NFS (Mixed fruit juices)", "Orange juice, frozen concentrate, with calcium and vit. D added, diluted", "Grape juice, canned or bottled,

unsweetened, with added vitamin C", and "Orange juice, frozen concentrate, unsweetened, diluted"). Coconut water is not counted as juice by ASA24-Canada-2016 but was treated as 100% fruit juice for this analysis. 2) Mixed Items included items that contain non-100% fruit juice sources of free sugar or 100% fruit juice plus other sources of free sugar. Items in this category include "Fruit smoothie drink, made with fruit or fruit juice only (no dairy products)", "Sauce, fruit (All fruits)", "Juice drink, fruit, ready-to-drink", "Fruit smoothie drink, NFS", "Fruit cocktail (peach, pear, apricot, pineapple, cherry, grape), canned, juice pack, solids and liquid", "Drink, fruit punch, vitamin C added, ready-to-drink", "Fruit flavored drink, low calorie, with high vitamin C", "Peach, canned halves or slices, juice pack, solids and liquid", "Lemon juice, raw", "Hummus, homemade", "Sauce, plum, ready-to-serve", "Macaroni or pasta salad", "Dessert, frozen, sherbet, orange", "Pad Thai with meat", "Macaroni or pasta salad with cheese", "Sweet and sour chicken", "Lemonade, frozen, diluted with water", "Fruit syrup (Strawberry, Blueberry)", "Light ice cream, creamsicle or dreamsicle (formerly ice milk)", "Sweets, jellies", "Macaroni or pasta salad W/ MAYONNAISE-TYPE SALAD DRESSING (INCLUDE MIRACLE WHIP)", "Sweets, fruit butters, apple".

[3]An in -house 'Grams Sugar Per Cup Fruit Juice' database was populated using ASA24 total sugar data for each fruit juice, e.g., "Apple juice, canned or bottled, added vitamin C" contains 24.05g / cup eq.
