# Supplementary material for: Free sugar intake from snacks and beverages in Canadian preschool- and toddler-aged children: a cross-sectional study
Source: BMC Nutr. 2023 Mar 8;9:44. doi: 10.1186/s40795-023-00702-3 (PMC9996946; doi:10.1186/s40795-023-00702-3)
Supplement: Supplementary file 2 — Additional file 2. Categories and subcategories of snacks and beverages (Adapted from Bernstein et al., 2016). [file 40795_2023_702_MOESM2_ESM.pdf]

Additional File 2: Categories and subcategories of snacks and beverages (Adapted from Bernstein et al., 2016)[1]

Food Group	Subcategory
Bakery Products	Baked Breakfast
	Baked Desserts
	Bread Products
	Cake
	Cereal/Granola Bars
	Cookies
	Pies, Tarts, Cobblers, Crisps
Beverages	Sugar-Containing Beverages[2]
	Unsweetened Milk
	Flavored Milk
	Plant-based beverages
	Fruit Juice
	Fruit Drinks
	Yogurt Beverage
	Smoothies
	Water
	Hot Beverages
	Regular Soft Drinks
	Diet Soft Drinks
	Sports Drinks
	Vegetable Drinks
	Energy Drinks
Cereals and Grain Products	Hot Breakfast Cereal
	Other Cereals and Grains
	Ready-to-eat breakfast cereal
Dairy Products and Alternates	Cheese
	Flavored yogurt
	Plain yogurt
	Cream cheese
Frozen Desserts	
Fats, Oils and Vinegars	Butter, margarine, oils
	Mayonnaise
Fruit	Raw fruit
	Fruit puree
	Dried fruit
	Fruit leather
	Canned fruit
	Frozen fruit
Meats, Eggs and Substitutes	Deli meats
	Eggs
	Meat and poultry

Mixed Dishes, Sides and Entrees	Beans
	Taco
	Soup
	Pizza
	French fries/hash browns
	Seafood salad
	Dumplings
	Pot pie
	Spaghetti
Nuts and Seeds	Butters, pastes and creams
	Nuts and seeds
	Nuts and seeds, Not For Snacking
Oral Nutrition Supplement	
Sauces, Dips and Condiments	Condiments
	Dips
	Sauces
Savory Snacks	Chips, corn and rice snacks
	Crackers
	Ethnic snacks
	Popcorn
	Pretzels
Confectionary and Sweet Condiments	Confectionary
	Sweet condiments
Vegetables and Legumes (except fried potatoes)	Raw vegetables
	Cooked vegetables and legumes
	Pickled vegetables
	Salad

[1] Bernstein JT, Schermel A, Mills CM, L'Abbé MR. Total and free sugar content of Canadian prepackaged foods and beverages. Nutrients 2016;8:582.
[2] Sugar-containing beverages included beverages with sugars added during processing + 100% fruit juice.
