# Supplementary figures and images for: Free sugar intake from snacks and beverages in Canadian preschool- and toddler-aged children: a cross-sectional study
Source: BMC Nutr. 2023 Mar 8;9:44. doi: 10.1186/s40795-023-00702-3 (PMC9996946; doi:10.1186/s40795-023-00702-3)

### Additional File 3: Participant flow chart

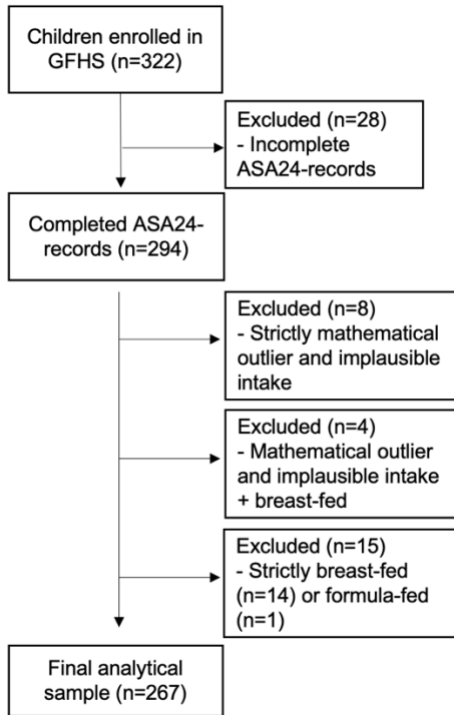

Supplement: Supplementary file 3 — Additional file 3. Participant flow chart. [file 40795_2023_702_MOESM3_ESM.pdf]
