# Supplementary material for: Free sugar intake from snacks and beverages in Canadian preschool- and toddler-aged children: a cross-sectional study
Source: BMC Nutr. 2023 Mar 8;9:44. doi: 10.1186/s40795-023-00702-3 (PMC9996946; doi:10.1186/s40795-023-00702-3)
Supplement: Supplementary file 4 — Additional file 4. Snack intake by major and minor snack categories and their contribution to free sugar energy intake among children. [file 40795_2023_702_MOESM4_ESM.pdf]

Additional File 4: Snack intake by major and minor snack categories and their contribution to free sugar energy intake among children[1]

Snack Category	Number of Children (Percent)			Children's %TE from FS Mean (95% CI)
	≥1	1	≥2	
**ALL SNACKS**	260 (97)	17 (6)	243 (91)	4.0 (3.5 – 4.5)
**BEVERAGE SNACKS**	121 (45)	81 (30)	40 (15)	1.8 (1.3 – 2.3)
Sugar-Containing Beverages[2]	53 (20)	41 (15)	12 (4)	4.1 (3.2 – 4.9)
Fruit Juice	20 (7)	~	~	-
Smoothies	10 (4)	~	~	-
Plain Milk	73 (27)	49 (18)	24 (9)	0
**FOOD SNACKS**	257 (96)	24 (9)	233 (87)	3.2 (2.8 – 3.6)
**Candy and Sweet Condiments**	56 (21)	50 (19)	6 (2)	3.0 (2.3 – 3.8)
Confectionary	41 (15)	36 (13)	5 (2)	2.8 (2.3 – 3.3)
Sweet Condiments	16 (6)	~	~	-
**Bakery Products**	148 (55)	108 (40)	40 (15)	2.4 (2.1 – 2.7)
Baked Desserts	15 (6)	~	~	-
Bread Products	57 (21)	50 (19)	7 (3)	1.9 (1.3 – 2.5)
Cake	7 (3)	~	~	-
Cereal/Granola Bars	42 (16)	~	~	2.4 (2.1 – 2.7)
Cookies	55 (21)	~	~	2 (1 – 2)
**Dairy Products and Alternates**	94 (35)	69 (26)	25 (9)	1.1 (0.7 – 1.6)
Cheese	46 (17)	39 (15)	7 (3)	0
Flavored Yogurt	40 (15)	~	~	2.7 (1.9 – 3.4)
Plain Yogurt	10 (4)	~	~	0
**Savory Snacks**	111 (42)	93 (35)	18 (7)	0.3 (0.2 – 0.5)
Chips, Corn and Rice Snacks	33 (12)	~	~	0
Crackers	64 (24)	58 (22)	6 (2)	0.4 (0.3 – 0.5)
Popcorn	16 (6)	~	~	-
**Fruits**	191 (72)	86 (32)	105 (39)	0.5 (0.3 – 0.8)
Raw Fruit	172 (64)	96 (36)	76 (28)	0
Fruit Puree	28 (10)	~	~	-
Dried Fruit	16 (6)	~	~	-
Fruit Leather	11 (4)	~	~	-

**Vegetables and Legumes (except fried potatoes)**	44 (16)	30 (11)	14 (5)	0.01 (-0.01 – 0.03)
Raw vegetables	35 (13)	21 (8)	14 (5)	0
**Cereals and Grain Products**	29 (11)	24 (9)	5(2)	-
**Nuts and Seeds**	42 (16)	35 (13)	7 (3)	0.1 (0.1 – 0.2)
Nuts and Seeds including trail mix	24 (9)	~	~	-
Butters, Pastes and Creams	19 (7)	18 (7)	1 (0.4)	-
**Mixed Dishes, Sides and Entrees**	16 (6)	~	~	-
**Frozen Desserts**	12 (4)	~	~	-
**Meats, Eggs and Substitutes**	11 (4)	~	~	-

[1] This table summarizes the number and percent of children 1.5-5 years who consumed different snack categories, relative to the total number of children (n=267). Percent was calculated to identify the proportion of children who consumed snack categories at least once (≥1) over 24-h, then calculated for the more discrete categories of one time (1) or two or more times (≥2). Not all children were reported to consume a snack (n=7). Mean children's %TE from FS was calculated for categories that were consumed by ≥30 children. For these calculations, only data from children who consumed each respective category were used (i.e., data from children who did not consume the snack category over 24-h were not included in the mean). Generalized estimating equations were used to account for any dependence between sibling participants (23). For categories consumed by <30 children, mean %TE from FS was not calculated and was instead denoted with a dash. Categories consumed and/or breakdowns were not reported and were denoted as ~ whenever <5 children were identified. Categories consumed by <10 children were removed including (Bakery Products: baked breakfasts (n=6), pies, tarts, cobblers, and crisps (n<5)), (Beverages: flavored milk (n=9), plant-based beverages (n=9), fruit drinks (n<5), yogurt beverages (n=5), hot beverages (n<5), sports drinks (n<5)), (Dairy Products and Alternates: cream cheese (n=6)), (Fruits: canned fruit (n=1), frozen fruit (n=6)), (Meats, Eggs, and Substitutes: Deli Meats (n<5), Eggs (n<5), Meat and Poultry (n=6)), (Nuts and Seeds: Nuts and seeds, not for snacking (n=5), (Savory Snacks: ethnic snacks (n<5), pretzels (n=9), (Mixed Dishes, Sides and Entrees: Taco (n<5), soup (n<5), pizza (n<5), french fries/hash browns (n=7), sandwich (n<5), seafood salad (n<5), dumplings (n<5), pot pie (n<5), spaghetti (n<5)), (Vegetables: cooked vegetables and legumes (n=9), pickled vegetables (n<5), and salad (n<5)).

[2] Sugar-containing beverages included beverages with sugars added during processing + 100% fruit juice. FS, Free Sugar; TE, Total Energy
