# Supplementary material for: Free sugar intake from snacks and beverages in Canadian preschool- and toddler-aged children: a cross-sectional study
Source: BMC Nutr. 2023 Mar 8;9:44. doi: 10.1186/s40795-023-00702-3 (PMC9996946; doi:10.1186/s40795-023-00702-3)
Supplement: Supplementary file 5 — Additional file 5. Guelph Family Health Study consortium members. [file 40795_2023_702_MOESM5_ESM.pdf]

Guelph Family Health Study Consortium Members

This document lists past and present members (faculty investigators, postdoctoral researchers, students, and staff) who have contributed to the Guelph Family Health Study as of January 1st, 2023.

Affiliations

- HHNS (Department of Human Health and Nutritional Sciences, University of Guelph, Guelph, Ontario, Canada, N1G 2W1)
- FRAN (Department of Family Relations and Applied Nutrition, University of Guelph, Guelph, Ontario, Canada, N1G 2W1)
- M&S (Department of Mathematics and Statistics, University of Guelph, Guelph, Ontario, Canada, N1G 2W1)
- MCB (Department of Molecular Biology, University of Guelph, Guelph, Ontario, Canada, N1G 2W1)
- BIOM (Department of Biomedical Sciences, University of Guelph, Guelph, Ontario, Canada, N1G 2W1)

GFHS Current Faculty Investigators

Dr. David Ma, Director (HHNS)
Dr. Jess Haines, Co-Director (FRAN)
Dr. Andrea Buchholz (FRAN)
Dr. Alison Duncan (HHNS)
Dr. Gerarda Darlington (M&S)
Dr. Lori Ann Vallis (HHNS)
Dr. Jen Monk (HHNS)
Dr. Dalia El-Khoury (FRAN)
Dr. Clara Cho (HHNS)

GFHS Past Faculty Investigators

Dr. David Mutch (HHNS)
Dr. Emma Allen-Vercoe (MCB)
Dr. Genevieve Newton (HHNS)
Dr. Lawrence Spriet (HHNS)
Dr. Paula Brauer (FRAN)

Postdoctoral Researchers

Ana Carolina Leme (FRAN)
Jessie Burns (HHNS)
Julia Gruson-Wood (FRAN)
Maude Perreault (FRAN)
Raphaelle Jacob (FRAN)

PhD Students

Amar Laila (FRAN)
Anisha Mahajan (HHNS & FRAN)
Becky Breau (HHNS)
Elie Chamoun (HHNS)

Hannah Coyle-Asbil (HHNS)
Jessie Burns (HHNS)
Katherine Eckert (FRAN)
Kathryn Walton (FRAN)
Lisa Tang (FRAN)
Rachel Ackford (HHNS)
Rahbika Ashraf (HHNS)
Sabrina Douglas (FRAN)
Sandhya Sahye (HHNS)
Tamara Petresin (FRAN)
Valerie Hruska (HHNS)
Zeinab Jafari (FRAN)

MSc Thesis Students
Alyssa Ramuscak (FRAN)
Amelie Bosse (FRAN)
Angela Wallace (FRAN)
Carley O'Kane (FRAN)
Darla Barsse Maldonado (FRAN)
Fatima Chleilat (HHNS)
Hannah Coyle-Asbil (HHNS)
Jessica Yu (HHNS)
Julia Broad (FRAN)
Keira De Bruijn (M&S)
Kira Jewell (FRAN)
Laura Wilson (FRAN)
Lisa Tang (FRAN)
Mackenzie Harris (FRAN)
Marciane Any (FRAN)
Mwalu Peeters (FRAN)
Nicholas Carroll (HHNS)
Nishita Sharif (M&S)
Sabrina Douglas (HHNS & FRAN)
Samantha Wong (FRAN)
Sarah Wedde (FRAN)
Valerie Hruska (FRAN)
Victoria Ambrose (FRAN)
MSc Coursework Students
Amar Leila (HHNS)
Animesh Vadaparti (M&S)
Becky Breau (HHNS)
Cicely Huinink (HHNS)
Dee Muszynski (HHNS)
Elie Chamoun (HHNS)
Hannah Whibbs (HHNS)
Jaimie Hogan (HHNS)
Jonathan Gillmore (HHNS)
Julia Mirotta (HHNS)

Justin Sheremeta (HHNS)
Laura MacRae (HHNS)
Myriam Philippi (HHNS)
Nicole Topakas (HHNS)
Olivia Landon (HHNS)
Patricia Acosta (HHNS)
Rahbika Ashraf (HHNS)
Rebecca Lewis (HHNS)
Robyn Barefoot (HHNS)
Victoria Srbely (HHNS)

Undergraduate Students – Research Assistants, 4th year thesis projects
Amar Leila (HHNS)
Andrea DeFulvis (HHNS)
Angel Anny Sousa Liu (HHNS)
Angela Picot (HHNS)
Annika Vels (HHNS)
Bonnie (Chi-En) Shao (FRAN)
Charlotte Jones (FRAN)
Claire Mazzia (HHNS)
Emily Farr (FRAN)
Erin Smith (HHNS)
Flora Zhang (FRAN)
Gitasha (Tasha) Eland (M&S)
Hannah Coyle-Asbil (HHNS)
Hannah Leung (FRAN)
Imtisal Janjua (HHNS)
Jaimie Hogan (HHNS)
Jake Chaput (HHNS)
Jane Flapper (FRAN)
Jessica Watterworth (FRAN & HHNS)
Jessica Yu (HHNS)
Joy MacKay (FRAN)
Kasia Kowanda (HHNS)
Katarina Osojnicki (HHNS)
Kate Nicholson (HHNS)
Katherine Adamski (FRAN)
Kim Gotera (M&S)
Lara Haines-Love (FRAN)
Laurie Matthews (HHNS)
Lisa Wedel (HHNS)
Maggie Bolton (M&S)
Nadia Browning (FRAN)
Nicholas Carroll (HHNS)
Owen Krystia (FRAN)
Rayann Henderson (HHNS)
Sabrina Turini (HHNS)
Sanny Li (FRAN)

Sarah Shpurko (FRAN)
Sarah Tiessen (FRAN)
Sarah Wedde (FRAN)
Shearin Fahandazh (FRAN)
Sophia Hou (FRAN)
Sophie Lloyd (FRAN)
Sunny (Tianxue) He (FRAN)
Yinuo (Amanda) Yang (FRAN)

Undergraduate Volunteers
Andrea Yazer (HHNS)
Anna Markov (FRAN)
Bridget Coyle-Asbil (BIOM & HHNS)
Brock Tompkins (HHNS)
Claire Mazzia (HHNS)
Erin Smith (BIOM & HHNS)
Hannah Mosiondz (BIOM & HHNS)
Hayley Wilson (BIOM & HHNS)
Isaac Bell (FRAN)
Jenna Westra (FRAN)
Jessica Durham (FRAN)
Joann Thangthong (FRAN)
Katarina Osojnicki (HHNS)
Kate Nicholson (HHNS)
Kelsey Sobkowich (FRAN)
Kendall Wilson (HHNS)
Kerrington Howell (FRAN)
Mackenzie Bailey (HHNS)
Melanie Beck (FRAN)
Melissa Kwan (FRAN)
Nicole Middegaal (FRAN)
Nicole Pandolfi (FRAN)
Olivia Roberge (FRAN)
Renee Ouellette (FRAN)
Rose Holub (FRAN)
Sara Riazi (HHNS)
Sijie Wen (BIOM & HHNS)
Sophia Hou (FRAN)
Sydney Winters (FRAN)
Victoria Friscioni (FRAN)
Zoe Todd (FRAN)

Secondary School Students
Helen Xu (FRAN)
Isaac Bell (FRAN)
Isabel Carsjens (HHNS)

Staff Support

Adam Sadowski (FRAN)
Alex Carriero (HHNS)
Alicia Martin (FRAN)
Alyssa Vets (FRAN)
Amar Laila (FRAN)
Amina Saher (FRAN)
Aneesh Viswamohan (FRAN)
Ang Picot (HHNS)
Angela Annis (HHNS & FRAN)
Angela Wallace (FRAN)
Avery Zenker (FRAN)
Bridget Coyle-Asbil (HHNS)
Carley O'Kane (FRAN)
Clara Gilliland (FRAN)
Erin Smith (HHNS)
Flora Zhang (FRAN)
Hilary Lo (HHNS & FRAN)
Jacob Schwartz (FRAN)
Jaimie Hogan (HHNS)
Jessica Yu (HHNS)
Julia Broad (FRAN)
Julia DeMiglio (FRAN)
Julia Mirotta (HHNS)
Julia Shannon (HHNS)
Kamel Omar (HHNS)
Lisa Wedel (HHNS)
Lyn Hillyer (HHNS)
Madeline Nixon (HHNS)
Madison Rodrigue (HHNS)
Michael Prashad (FRAN)
Nadia Browning (FRAN)
Nicholas Carroll (FRAN)
Sabrina Douglas (HHNS)
Sanny Li (FRAN)
Sarah Wedde (FRAN)
Shannon Pare (FRAN)
Shearin Fahandazh (FRAN)
Sophia Hou (FRAN)
Sunny (Tianxue) He (FRAN)
Tory Ambrose (FRAN)
Zach Ribau (HHNS)
